# Development and Validation of Prognostic Nomograms Based on Gross Tumor Volume and Cervical Nodal Volume for Nasopharyngeal Carcinoma Patients With Concurrent Chemoradiotherapy

**DOI:** 10.3389/fonc.2021.682271

**Published:** 2021-06-28

**Authors:** Cui-Dai Zhang, Mei Li, Ying-Ji Hong, Ze-Man Cai, Kai-Chun Huang, Zhi-Xiong Lin, Zhi-Ning Yang

**Affiliations:** ^1^ Department of Radiation Oncology, Cancer Hospital, Shantou University Medical College, Shantou University, Shantou, China; ^2^ Nasopharyngeal Carcinoma Research Center, Cancer Hospital, Shantou University Medical College, Shantou University, Shantou, China; ^3^ Shantou University Medical College, Shantou University, Shantou, China

**Keywords:** nasopharyngeal neoplasm, nomogram, chemoradiotherapy, prognosis, least absolute shrinkage and selection operator, decision curve analysis

## Abstract

**Purpose:**

Our study aimed to establish and validate prognostic nomograms based on gross tumor volume (GTV) and cervical nodal volume (CNV) for nasopharyngeal carcinoma (NPC) patients treated with two cycles of concurrent chemoradiotherapy (CCRT).

**Methods:**

From 2012 to 2015, 620 eligible patients who received radical treatment at the Cancer Hospital of Shantou University Medical College were recruited for a nomogram study. Variables were determined in a training set of 463 patients from 2012 to 2014 by X-tile analysis, univariate and multivariate Cox proportional hazard analyses, and the least absolute shrinkage and selection operator (LASSO). Another cohort of 157 patients in 2015 was validated with bootstrap resampling. The concordance index (C-index) and calibration curves were applied to assess its predictive discriminative and accuracy ability, while decision curve analysis (DCA), X-tile analysis and Kaplan–Meier curve for clinical application.

**Results:**

Independent prognostic variables for overall survival (OS) were age, GTV, CNV, cranial nerve, positive cervical lymph node laterality below the caudal border of cricoid cartilage (LNBC), and were selected for the nomogram. Optimal prognostic factors including Karnofsky performance status (KPS), age, GTV, CNV, LNBC were incorporated in the nomogram for progression-free survival (PFS). In the training set, the C-index of our nomograms for OS and PFS were 0.755 (95% CI, 0.704 to 0.807) and 0.698 (95% CI, 0.652 to 0.744). The calibration curve showed good agreement between nomogram-predicted and actual survival. DCA indicated that our nomograms were of clinical benefit.

**Conclusion:**

Our nomograms are capable of effective prognostic prediction for patients with NPC.

## Introduction

NPC is an endemic malignant tumor that has the highest incidence reported in Southern China and Southeast Asia (1). Combined chemotherapy and radiotherapy have been the current standard of treatment for NPC. Overall prognosis has furtherly improved in recent years (1–3). At present, the American Joint Committee on Cancer/Union for International Cancer Control (AJCC/UICC) TNM staging system is used widely to predict prognosis for NPC. However, patients who at the same TNM stages and with identical or similar treatment regimens still have diverse survival rates (4). This indicates the limitations of TNM staging and shows it is not adequate enough to predict prognosis. There is increasing evidence that GTV (2), CNV (3), Epstein–Barr virus (EBV) DNA copy number (5), tumor-infiltrating lymphocytes (TILs) (6) and lactate dehydrogenase (LDH) (7) are significant prognostic factors that might serve as indicators to develop more effective, accurate and applicable prediction models.

Nomograms play an increasingly important role in predicting prognosis for various kinds of cancers by incorporating assorted effective factors to quantify individual risk (8). Previous studies have developed nomograms for patients with NPC consisting of clinical factors (5, 6, 9), hematological biomarkers (5, 6, 9), anatomic characteristics (5) or therapeutic regimens (6, 9). However, no study has constructed a nomogram for patients with NPC based on GTV and CNV up to now. The aim of our study is to create effective prognostic nomograms for NPC patients with two cycles of CCRT based on GTV and CNV.

## Materials and Methods

### Patients

A total of 1,370 NPC patients treated at the Cancer Hospital of Shantou University Medical College from 2012 to 2015 were recruited. The main inclusion criteria consisted of the following: (i) primary NPC patients confirmed by histopathology and with integrated and adequate clinical data; (ii) received two cycles of CCRT without induction chemotherapy (IC) or adjuvant chemotherapy (AC); (iii) no distant metastasis before or during treatment; and (iv) absence of other severe diseases or cancer. Of the total patients, 620 patients were selected in our retrospective study and divided into a training and validation set, while 750 patients were excluded. The training set included 463 patients who received complete treatment from 2012 to 2014. The validation set contained 157 patients who were treated in 2015. TNM stages were restaged, in this 620-case cohort, based on the eighth edition of the AJCC/UICC staging system.

### Clinical Data Collection Process

Five variables of clinical information were extracted from the electronic medical and clinical records, and included KPS, sex, age, pathological features and chemotherapy regimens. GTV comprised both retropharyngeal nodes and gross tumor volume. GTV and CNV were calculated by 3D treatment planning software (Eclipse, version 10.0, Varian Radiation Oncology System). Fifty-six anatomically involved sites of the tumor were extracted from magnetic resonance imaging (MRI) and a 3D treatment planning software such as GTV, and included the oropharynx, nasal cavity, levator veli palatini, tensor veli palatini, medial pterygoid, lateral pterygoid, prevertebral muscles, parapharyngeal space, retropharyngeal space, prevertebral space, sphenoid bone, pterygoid process, clivus, paranasal sinus, cavernous sinus, cranial nerve, and cervical vertebra. Twenty-six variables of cervical lymph nodes were recorded, such as CNV, the greatest dimension, nodal level and laterality, nodal grouping, and extracapsular spread.

### Therapeutic Regimens

All patients received two cycles platinum-based chemotherapy concurrently with radiotherapy. The concurrent chemotherapy regimens included PF (cisplatin 25–30 mg/m^2^/d days 1–3 or nedaplatin 80–100 mg/m^2^, plus fluorouracil 800–1000 mg/m^2^/d, days 1–5), which accounted for 63.9% and TP (docetaxel 75–80 mg/m^2^ day 1 or paclitaxel 135–175 mg/m^2^ day 1 plus cisplatin 25–30 mg/m^2^/d days 1–3), which accounted for 36.1%. The chemotherapy was administered every three weeks for two cycles. We delineated the target volume on the basis of the International Commission on Radiation Units and Measurements Reports 50 and 62. The high-risk region was defined as CTV1 and the low-risk region was known as CTV2. The dose of the planning target volume was 69.9 Gy in 30 fractions or 70.4 Gy in 32 fractions. The dose of CTV1 and CTV2 were 60 and 54 Gy in the identical number of fractions, respectively.

### Follow-Up

We followed up patients with NPC through the clinic attendance records at the outpatient department or phone contact every 3–6 months during the first 3 years, every 6–12 months for years 4–5, and annually thereafter until death. Patient follow-up visits included fiberoptic nasopharyngoscopy, MRI of the head and neck, abdominal sonography or CT, CT of the chest, and a bone scan. We deemed OS to be the time from the initial date of diagnosis to the date of death from any cause or date of censor (July 31, 2020). PFS was the duration from the initial date of diagnosis to the date of metastasis, recurrence, death from any causes or date of censor (July 31, 2020).

### Statistical Analysis

**Figure 1:**
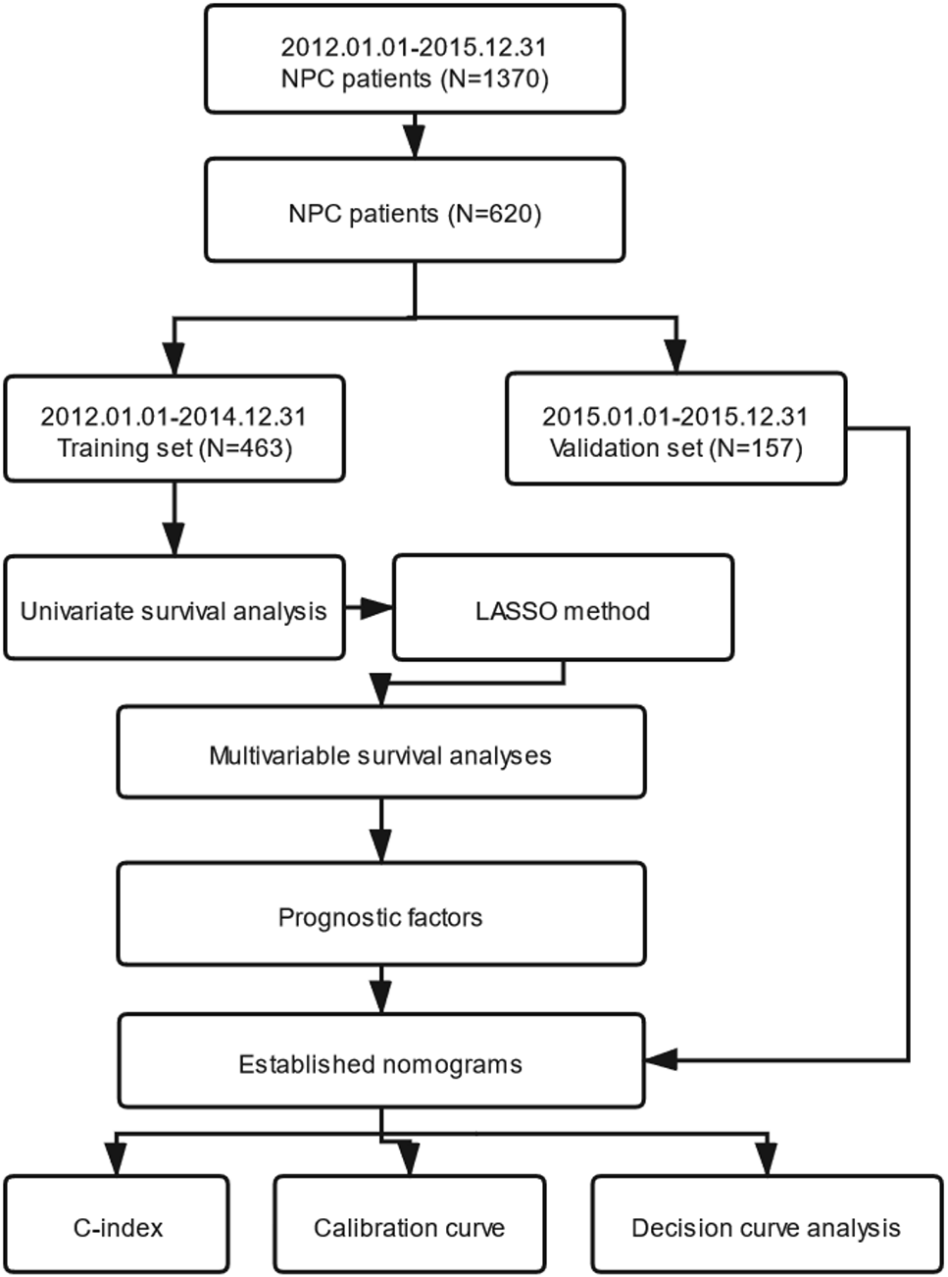
The entire analysis procedure. NPC, nasopharyngeal carcinoma; LASSO, least absolute shrinkage and selection operator; C-index, concordance index.

In this study, OS was the primary endpoint and PFS was the second. We transformed continuous variables into categorical variables, using the X-tile program, which were designed for cut-point optimization (Version 3.6.1, Yale University) (10). IBM SPSS statistical software version 24.0 (IBM, Armonk, NY, USA) was used for statistical analysis. Cox’s proportional hazard regression model with conditional forward stepwise variable selection, depending on the training set, was applied for univariate survival analysis. We selected every variable with level of P <0.05 (two sided), which indicated that it had a statistically significant difference after univariate survival analysis. Secondly, the LASSO method, which was applicable for choosing a biomarker in high dimensional data, was utilized to reselect the most significant factors. The LASSO method can ameliorate the interpretability and accuracy of regression models through regularization (11, 12). Thirdly, the optimal prognostic variables selected by the LASSO method were entered into multivariable survival analyses performed using the Cox’s proportional hazard regression model with conditional forward stepwise variable selection subsequently. Fourthly, nomograms were established through the rms package in R software (R version 3.6.1) according to the independent prognostic variables selected by the multivariable survival analyses. The nomograms for OS and PFS were compared with the eighth edition of the AJCC/UICC staging system by evaluating the C-index. Nomogram-predicted and actual absence of progression rate as well as survival rate were detected in the calibration curve. We performed bootstraps with 1,000 resamples to avoid over-optimism according to Transport Reporting of a Multivariable Prediction Model for Individual Prognosis of Diagnosis (TRIPOD) statement (13). Next, DCA was used for the clinical feasibility and benefits of the nomogram models (14, 15). Furthermore, patients in the training set were grouped into three risk groups (low-risk, intermediate-risk and high-risk groups) according to the total scores calculated by our nomograms for OS and PFS. The X-tile program was utilized for the first-rank cut-off value of the risk score. Finally, we verified the predictive ability through Kaplan–Meier survival curve analysis according to cut-off points of the risk stratification determined by the X-tile program. The procedure of this study is illustrated in **Figure 1**.

## Results

### Baseline Characteristics and Prognosis

Survival analyses were performed on 463 patients from the training set and 157 patients from the validation set. Patient demographic and clinical characteristics are illustrated in **Table 1**. Median and average follow-up periods were 70.0 and 68.8 months for patients in the training set, and 59.0 and 56.0 months in the validation set, respectively. Moreover, the 3- and 5-year OS in the training set were 88.3 and 84% and that were 94.1 and 88.2% in the validation set, respectively (P=0.152). The 3- and 5-year rates for PFS were 83.6 and 75.3% in the training set and that were 83.1 and 73.3% in the validation set, respectively (P=0.846).

**Table 1:** Clinical features of patients with nasopharyngeal carcinoma.

Characteristics	Training set(N = 463)	Validation set(N = 157)	P-value
	No. of patients(%)	No. of patients(%)	
Age(years)			
≤51	278 (60.0)	93 (59.2)	0.179
>51, ≤62	135 (29.2)	54 (34.4)	
>62	50 (10.8)	10 (6.4)	
Sex			
Male	327 (70.6)	127 (80.9)	**0.016**
Female	136 (29.4)	30 (19.1)	
KPS			
70	2 (0.4)	1 (0.6)	0.535
80	57 (12.3)	25 (15.9)	
90	399 (86.2)	128 (81.5)	
100	5 (1.1)	3 (1.9)	
Pathologic type			
Undifferentiated nonkeratotic carcinoma	244 (52.7)	100 (63.7)	0.057
Differentiated nonkeratotic carcinoma	200 (43.2)	52 (33.1)	
Others^a^	19 (4.1)	5 (3.2)	
T stage			
T1	48 (10.4)	17 (10.8)	**0.002**
T2	117 (25.3)	25 (15.9)	
T3	212 (45.8)	65 (41.4)	
T4	86 (18.6)	50 (31.8)	
N stage			
N0	10 (2.2)	8 (5.1)	0.224
N1	134 (28.9)	49 (31.2)	
N2	271 (58.5)	83 (52.9)	
N3	48 (10.4)	17 (10.8)	
Stage			
II	38 (8.2)	10 (6.4)	**0.009**
III	297 (64.1)	83 (52.9)	
IVA	128 (27.6)	64 (40.8)	
GTV (ml)			
≤24.9	224 (48.4)	64 (40.8)	0.173
>24.9, ≤58.9	150 (32.4)	63 (40.1)	
>58.9	89 (19.2)	30 (19.1)	
CNV (ml)			
≤10.9	191 (41.3)	61 (38.9)	0.707
>10.9, ≤41.4	218 (47.1)	74 (47.1)	
>41.4	54 (11.7)	22 (14.0)	
GD (cm)			
≤2.2	204 (44.1)	93 (59.2)	**0.001**
>2.2, ≤4.0	207 (44.7)	57 (36.3)	
>4.0	52 (11.2)	7 (4.5)	

a Others include squamous cell carcinoma, low differentiated squamous cell carcinoma and moderately differentiated squamous cell carcinoma. KPS, Karnofsky performance status; GTV, gross tumor volume; CNV, cervical nodal volume; GD, the greatest dimension of cervical lymph nodes. Bold text: statistical significance.

### Processing of Variables

We transformed continuous variables, including age, GTV, CNV and the greatest dimension of cervical lymph nodes (GD), into categorical variables using the X-tile program to determine the optimum cut-off values. First, in the training set, patients with NPC were divided into three subgroups according to their age: the first group (≤51, n = 278), the second group (>51, and ≤62, n = 135), and the third group (>62, n = 50). Second, patients were also assigned to three subgroups according to the value of the GTV: the first group (≤24.9 ml, n = 224), the second group (>24.9 ml, and ≤58.9 ml, n = 150), and the third group (>58.9 ml, n = 89). In addition, they were classified into three subgroups in light of the CNV value: the first group (≤10.9 ml, n = 191), the second group (>10.9 ml, and ≤41.4 ml, n = 218), and the third group (>41.4 ml, n = 54). Finally, they were divided into three subgroups in accordance with the GD: the first group (≤2.2 cm, n = 204), the second group (>2.2 cm, and ≤4.0 cm, n = 207), and the third group (>4.0 cm, n = 52). Likewise, in the validation set, patients were assigned to three subgroups, according to age, GTV, CNV and, by the same cut-off values as in the training set.

### Univariate Survival Analysis

From the training set, 87 variables (five variables for clinical information, 56 variables for gross tumor and 26 variables for cervical lymph nodes with metastasis) were entered into the univariate survival analysis (Cox’s proportional hazards regression model). As a result, a significant association of 32 variables (two clinical information variables, 18 gross tumor variables and 12 variables for cervical lymph nodes with metastasis) for OS were identified. A significant association of 28 variables (two clinical information variables, 13 gross tumor variables and 13 variables for cervical lymph nodes with metastasis) for PFS were selected. They are summarized in **Table S1**.

### LASSO Method to Reselect Optimal Prognostic Variables

**Figure 2:**
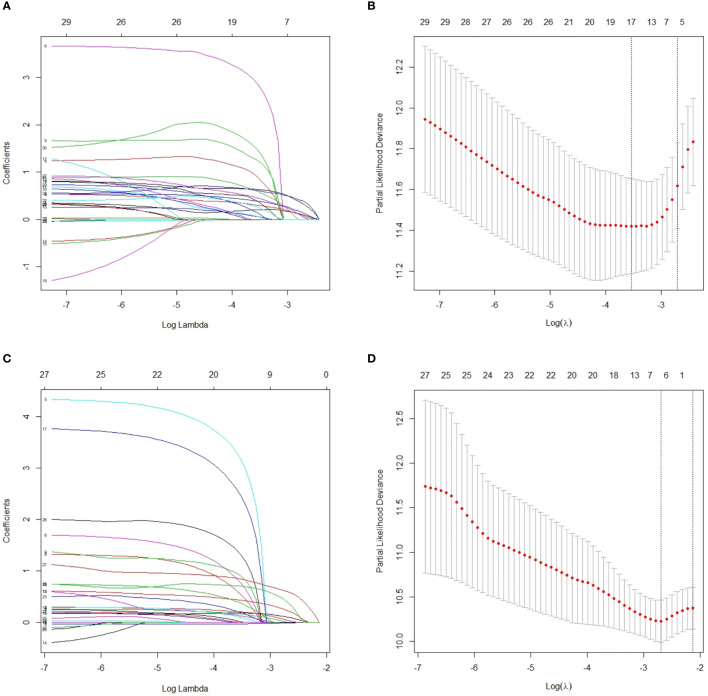
After initial screening by univariate analysis, selection of variables for OS and PFS were performed using the LASSO method with a logistic regression model. **(A)** Model coefficient trendlines of the 32 variables for OS. The profile graph was plotted by coefficients against the L1 norm (inverse proportional to log λ = −2.705). **(B)** Tuning parameter λ in the LASSO model. The parameter λ = 0.067 was selected under the minimum criteria. The vertical line was drawn at the value selected by 10-fold cross-validation, including optimized six non-zero coefficients. **(C)** Model coefficient trendlines of the 28 variables for PFS. The profile graph was plotted by coefficients against the L1 norm (inverse proportional to log λ = −2.688). **(D)** Tuning parameter λ in the LASSO model. The parameter λ = 0.068 was selected under the minimum criteria. The vertical line is drawn at the value selected by 10-fold cross-validation, including optimized seven non-zero coefficients. OS, overall survival; PFS, progression-free survival; LASSO, least absolute shrinkage and selection operator.

When lambda equaled 0.067 and log (λ) = −2.705, the residual sum of squares was shown to be the minimum for OS. Six optimal prognostic variables were identified, including age, GTV, CNV, cranial nerve, LNBC and lymph node laterality metastasis of level Vb/c (Vb/c-LN). When lambda equaled 0.068 and log (λ) = −2.688, the residual sum of squares was certified to be the minimum for PFS. This yielded 7 optimal prognostic variables containing KPS, age, GTV, CNV, cranial nerve, LNBC and positive bilateral cervical lymph node below the caudal border of the cricoid cartilage (BLNBC). We illustrate an elaborate description of the LASSO method in **Figure 2**.

### Multivariate Survival Analyses

We performed multivariate survival analyses with the Cox’s proportional hazards model to further verify the hazard ratio and coefficient for each variable reselected through the LASSO method. As a result, a significant association of five variables (age, GTV, CNV, cranial nerve, LNBC) for OS and five variables (KPS, age, GTV, CNV, LNBC) for PFS were selected that had a P <0.05. The results of the multivariate survival analyses are presented in **Table 2**.

**Table 2:** Multivariate Cox regression analyses for OS and PFS in NPC patients in the training set.

Variables	OS			PFS		
	P-value	HR	95% CI	P-value	HR	95% CI
KPS	–	–	–	**0.035**	0.954	0.913–0.997
Age (years)	**0.009**	1.367	1.081–1.727	**0.021**	1.320	1.042–1.672
GTV (ml)	**0.007**	1.403	1.097–1.794	**0.012**	1.371	1.071–1.756
CNV (ml)	**0.011**	1.418	1.083–1.857	**0.011**	1.417	1.083–1.853
Cranial nerve	**0.009**	2.006	1.194–3.372	0.075	1.663	0.951–2.908
Vb/c-LN	0.615	–	–	–	–	–
LNBC	**0.000**	2.836	1.735–4.64	**0.000**	2.973	1.813–4.874
BLNBC	–	–	–	0.085	–	–

OS, overall survival; PFS, progression-free survival; NPC, nasopharyngeal carcinoma; CI, confidence interval; KPS, Karnofsky performance status; GTV, gross tumor volume; CNV, cervical nodal volume; Vb/c-LN, lymph node laterality metastasis of level Vb/c; LNBC, positive cervical lymph node laterality below the caudal border of the cricoid cartilage; BLNBC, positive bilateral cervical lymph node below the caudal border of the cricoid cartilage. Bold text: statistical significance.

### Establishment of Nomograms

**Figure 3:**
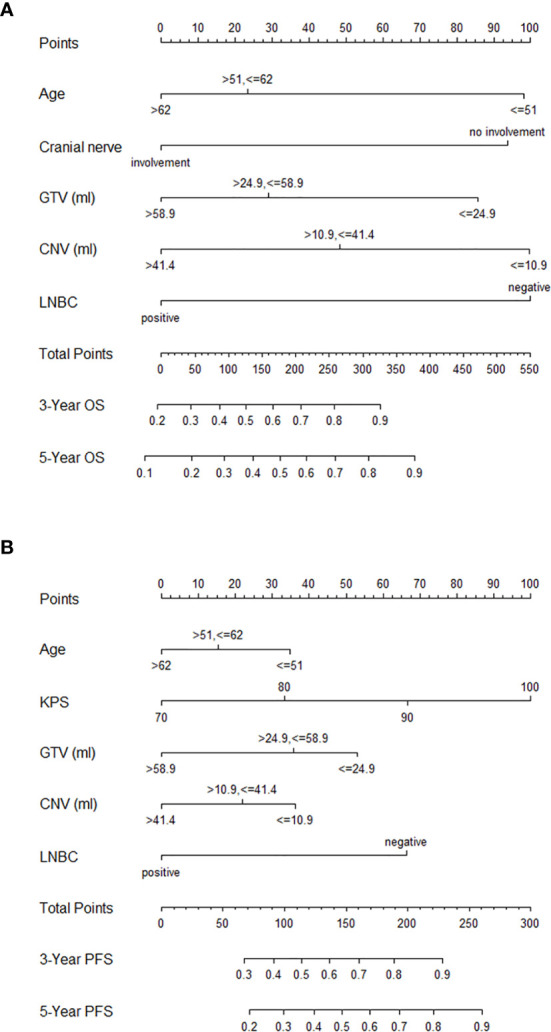
Nomograms for NPC patients with CCRT for OS **(A)** and PFS **(B)**. NPC, nasopharyngeal carcinoma; CCRT, concurrent chemoradiotherapy; OS, overall survival; PFS, progression-free survival; GTV, gross tumor volume; CNV, cervical nodal volume; LNBC, positive cervical lymph node laterality below the caudal border of the cricoid cartilage; KPS, Karnofsky performance status.

The above significant prognostic variables were used in our nomograms for OS and PFS, respectively. They are illustrated in **Figures 3A, B**. The nomogram for OS includes age, GTV, CNV, cranial nerve, and LNBC, whereas the nomogram for PFS contains KPS, age, GTV, CNV, and LNBC. We can predict the probability of the 3- and 5-year OS and PFS for an individual patient through these nomograms. The usefulness of a nomogram is that it maps the predicted probabilities into points on a scale from 0 to 100 in a user-friendly graphical interface. The total points accumulated by the various covariates correspond to the predicted probability for a patient (16). LNBC had the greatest impact on OS, followed by CNV, age, cranial nerve and GTV. At the meanwhile KPS had the greatest impact on PFS, followed by LNBC, GTV, CNV and age. By addition of the total score and locating it on the total point scale, we could draw a straight line down to determine the estimated probability of progression. It could predict the 3- and 5-year OS/PFS of NPC patients. For example, a 60-year-old NPC patient with cranial nerves involvement and positive LNBC, KPS = 90, GTV = 25 ml, CNV = 15 ml is assigned 100.6 total points according to the nomogram for OS, while he gets 139.4 total points according to the nomogram for PFS. For this patient, the estimated probability of 3- and 5-year OS is 42.5 and 32.0% and the 3- and 5-year PFS is 62.0 and 48.0%.

### Calibration and Validation of the Nomograms

**Figure 4:**
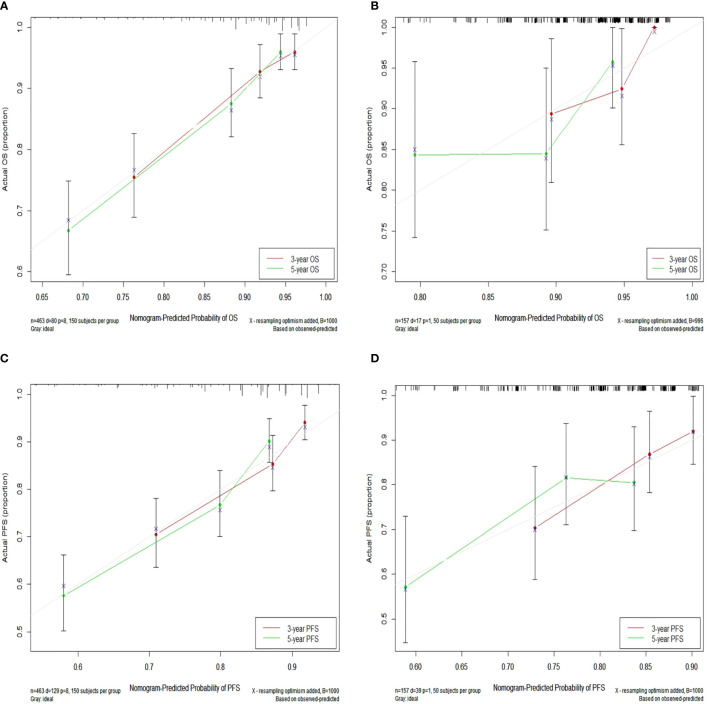
Calibration curves for 3- and 5-year OS in the training set **(A)** and calibration curves for 3- and 5-year OS in the validation set **(B)**; Calibration curves for 3- and 5-year PFS in the training **(C)** and validation **(D)** sets. Nomogram-predicted OS and PFS are plotted on the x-axis; actual OS and PFS are plotted on the y-axis. Dashed lines along the 45-degree line passing through the point of origin represent perfect calibration models in which predicted probabilities are identical to actual probabilities. OS, overall survival; PFS, progression-free survival.

In the training set, the bias-corrected C-index of the nomogram for OS was 0.755 (95% CI, 0.704 to 0.807), which is significantly better than that of the eighth edition of the AJCC/UICC TNM staging system (0.694, 95% CI, 0.638 to 0.749). Likewise, the C-index of the nomogram for PFS (0.698, 95% CI, 0.652 to 0.744) also performed better than the eighth edition of the AJCC/UICC TNM staging system (0.662, 95% CI, 0.614 to 0.710). In the validation set, our nomograms for OS and PFS still revealed better discrimination [C-index: 0.673, 95% CI, 0.551 to 0.796, and 0.638, 95% CI, 0.538 to 0.738, respectively] than the eighth edition of the AJCC/UICC TNM staging system [C-index: 0.591, 95% CI, 0.463 to 0.720, and 0.596, 95% CI, 0.508 to 0.684, respectively]. These results indicate that our nomograms have good discriminative ability. The detailed results are summarized in **Table 3**. Calibration curves indicated excellent agreement between our nomograms’ prediction and actual observation for 3- and 5-year OS and PFS in the training set (**Figures 4A, B**). Similarly, calibration curves revealed good agreement between our nomograms and actual observation for 3- and 5-year OS and PFS in the validation set (**Figures 4C, D**).

**Table 3:** The C-index of nomograms for OS and PFS in the training set and validation set.

Model for survival prediction	The training set		The validation set	
	C-index	95%CI	C-index	95%CI
OS nomogram	0.755	0.704–0.807	0.673	0.551–0.796
TNM classification(OS)^a^	0.694	0.638–0.749	0.591	0.463–0.720
PFS nomogram	0.698	0.652–0.744	0.638	0.538–0.738
TNM classification(PFS)^b^	0.662	0.614–0.710	0.596	0.508–0.684

^a^TNM classification (OS), OS nomogram based on TNM classification; ^b^TNM classification (PFS), PFS nomogram based on TNM classification. C-index, concordance index; OS, overall survival; PFS, progression-free survival; CI, confidence interval.

### Decision Curve Analysis

**Figure 5:**
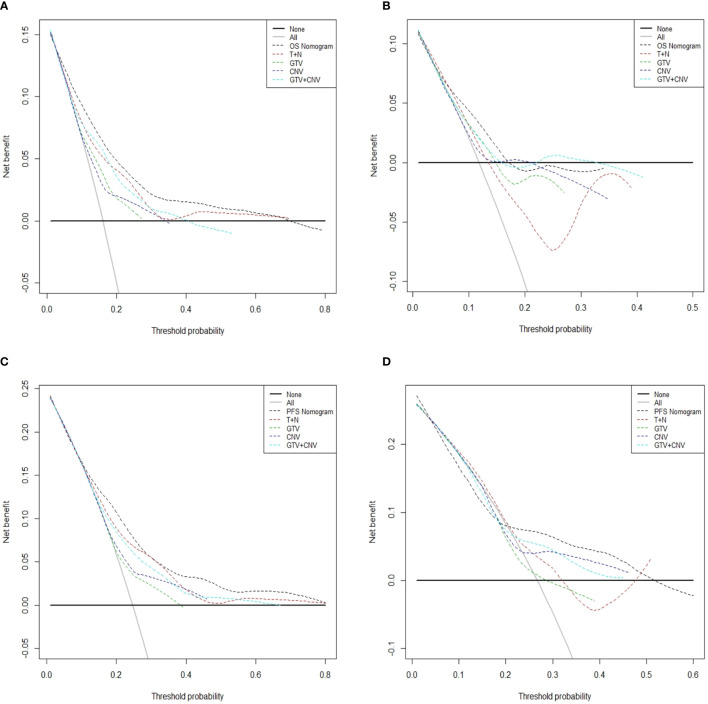
Decision curve analysis for the predicted-nomogram model of 5-year OS in the training **(A)** and validation **(B)** sets. Decision curve analysis for the predicted-nomogram model of 5-year PFS in the training **(C)** and validation **(D)** sets. OS, overall survival; PFS, progression-free survival; T, T stage; N, N stage; GTV, gross tumor volume; CNV, cervical nodal volume.

In **Figures 5**, the x-axis indicates the range of threshold probabilities, and the y-axis shows the net benefit, which is obtained by subtracting the harms (false positives) and summing the benefits (true positives) in DCA. DCA for the nomogram predictions for 5-year OS and PFS in the training and validation sets are presented in **Figures 5A–D**, respectively. DCA illustrates that our nomograms for OS and PFS (black dotted line) had the highest net benefit than that of T stage plus N stage (red dotted line). In addition, DCA was implemented to compare the clinical availability and benefits of the nomograms. The figures (**Figures 5A–D**) indicate that DCA curves of the nomograms for OS and PFS were superior to GTV alone, CNV alone and GTV plus CNV. Moreover, these figures not only illustrate that both GTV and CNV were prominent prediction and evaluation indicators, but also suggest that the combinations of GTV and CNV outperformed GTV or CNV alone.

### Risk Stratification

**Figure 6:**
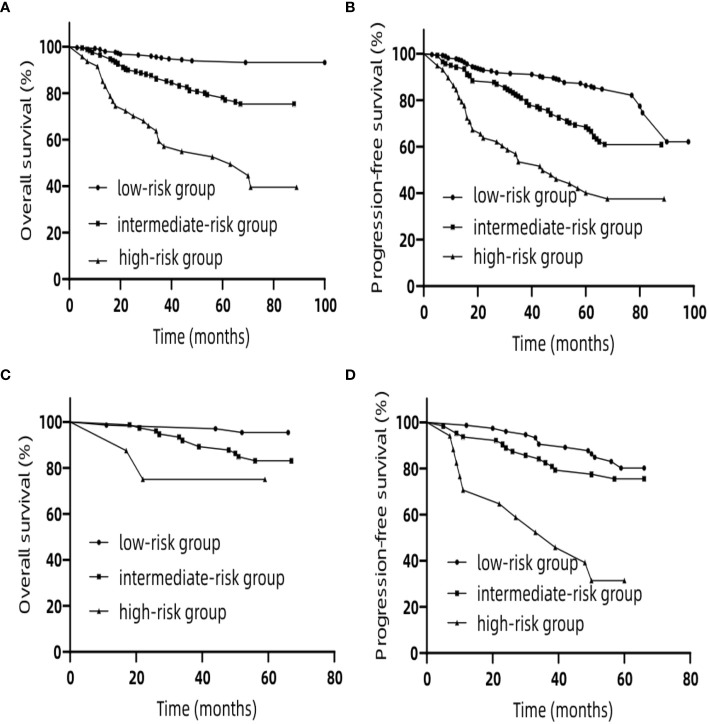
Kaplan–Meier curves for risk group stratification for OS **(A)** and PFS **(B)** in the training set. Kaplan–Meier curves for risk group stratification for OS **(C)** and PFS **(D)** in the validation set. OS, overall survival; PFS, progression-free survival.

In the training set, we classified patients into three subgroups on the basis of the cut-off values of the total points determined by the X-tile program: a low-risk group (>369.3 points, n = 208), an intermediate-risk group (>227.1 and ≤369.3 points, n = 208), and a high-risk group (≤227.1 points, n = 47) for OS. For PFS, patients were subdivided into three groups: a low-risk group (>209.3 points, n = 262), an intermediate-risk group (>157.4 and ≤209.3 points, n = 143), and a high-risk group (≤157.4 points, n = 58). In both the training and validation sets the Kaplan–Meier survival curves for OS and PFS were distinctly separate. In the training set, among the low-risk group, intermediate-risk group and high-risk group, the 5-year OS rates were 93.9, 77.9, and 52.6% (**Figure 6A**) (p <0.0001), respectively. Likewise, in the training set we also observed significant differences for PFS. Among the three risk groups, the 5-year PFS rates were 86.3, 68.5, and 40.1% (**Figure 6B**) (p <0.0001), respectively. In the validation set, among the low-risk group, intermediate-risk group and high-risk group, the 5-year OS rates were 95.4, 83.1, and 75.0% (**Figure 6C**) (p <0.05), respectively. Likewise, in the validation set we also observed significant differences for PFS. Among the three risk groups, the 5-year PFS rates were 80.1, 75.5, and 31.4% (**Figure 6D**) (p <0.0001), respectively. These results suggest that lower total points of the nomogram for OS are related to worse survival rate, and higher total points of nomogram for PFS are related to lower progression rate. These stratifications were able to efficiently discriminate the prognosis outcomes for the three risk subgroups mentioned above.

## Discussion

Research on developing nomograms for patients with NPC has been ongoing in recent years, but few papers have considered GTV and CNV. To the best of our knowledge, this is the first study to integrate GTV and CNV with MRI results of gross tumor lesions and cervical lymph nodes with metastasis to establish nomograms for OS and PFS in NPC patients with two cycles of CCRT. In our research, we developed and validated nomograms for predicting OS and PFS of patients with NPC. Five independent prognostic variables have been identified and incorporated into the nomogram for OS, containing age, GTV, CNV, cranial nerve, and LNBC. Our PFS-nomogram is comprised of five independent prognostic variables involving KPS, age, GTV, CNV, and LNBC. Our nomograms have been validated to have good discriminatory accuracy and can serve as convenient tools to predict prognosis for patients with NPC.

Some studies have reported that GTV and CNV significantly impact the prognosis of patients with NPC. Sze et al. suggested that GTV is a vital and independent prognostic feature for the local failure-rate, and the risk of local failure is estimated to increase by 1% for every 1 cm³ increase in GTV (17). Chen et al. demonstrated that the 5-year OS is reduced for patients with a large tumor volume (>50 ml), which is almost equal to that of T4 (2). Yuan et al. found that morphologic nodal volume is an important factor in prognostication and risk stratification in NPC (3). Based on our study, we suggest that a larger tumor and cervical nodal volume are significantly associated with a poor survival rate. We used DCA to further compare the prognostic prediction ability of our nomogram and the TNM staging system. The consequences of DCA illustrate that our nomograms offer higher net benefit compared with T stage plus N stage for forecasting OS and PFS. In other words, for predicting OS and PFS, the accurate prediction and independent discriminative ability of our nomograms are superior to the eighth edition of the TNM staging system.

Currently, no prior research has definitively evaluated CNV for clinical utility, but nomograms that include GTV as a crucial prognostic factor for NPC are available (18–22). However, these nomograms still have some limitations. First, patients with NPC in the retrospective studies accepted chemotherapy approaches that primarily incorporated IC, CCRT, AC, IC + CCRT, and CCRT + AC (18, 20–22). Different kinds of chemotherapy regimens have different correlations with prognosis. For instance, IC + CCRT improves the survival of patients with locoregionally advanced NPC (23). However, similar to randomized controlled trials, diversity in chemotherapy regimens can also result in differences in OS, even for tumors with similar stage (24, 25). Hence, our studies focused on patients with NPC who received two cycles chemotherapy regimens comprised of platinum-based chemotherapy in order to reduce the impact on prognosis because of the inevitable heterogeneity of chemotherapy regimens caused by the retrospective designs. Secondly, applicability of some nomograms has become outdated because they incorporate GTV and TNM stage based on the sixth or seventh editions of AJCC/UICC staging system (18–20). It is not the same to define T-stage in different editions of the AJCC/UICC staging system. For instance, when the medial pterygoid or lateral pterygoid is involved, T-stage is T2 according to the eighth edition while it is T4 based on the seventh edition. Our nomograms including GTV and CNV will not become obsolete even though the AJCC/UICC staging system will be continually updated.

KPS was incorporated in our nomogram for PFS since it has been shown to be involved in prognosis. Age was independent prognostic variable corelated with survival and progression, and it was included in our nomograms. Older patients with NPC have poorer survival because of increasing risk of comorbidities, lower tolerance to intensive therapies, and an impaired immune system (26). Therefore, this calls for a combination of multidisciplinary systemic treatment and oncologic nursing to further improve prognosis in older patients with NPC. Cranial nerve invasion has been shown to be a poor prognostic factor in NPC (27, 28). Liu et al. showed that NPC patients commonly show MRI-detected cranial nerve invasion, which is often seen in asymptomatic patients who experience more recurrences and distant metastases (28). LNBC was incorporated in our nomograms for OS and PFS. Li et al. showed that lower cervical lymph node metastasis (IV, Vb and supraclavicular regions) is an independent prognostic factor that affects survival (29). Our study concurs with these findings.

Several limitations should be noted in our study though our nomograms have good discriminatory accuracy. First, we did not take into account additional factors related to prognosis for NPC, such as EBV DNA copy number before treatment, TILs, and LDH. These factors will be potentially incorporated with GTV, CNV and anatomic structures in future revisions. Second, our data set was relatively small, only from a single center study. To further improve these nomograms, multicenter studies are warranted. Third, we cannot eliminate the influence of selection bias on outcomes suggested by us because of the retrospective nature of the study. Prospective studies should be considered to improve our nomograms. Fourth, the follow-up time for some patients in the validation set was less than 5 years, which may result in better prognosis prediction than actual prognosis. A continuous follow-up is necessary. The possible difficulties for wide diffusion of our nomograms in clinical daily practice might be that our nomograms are limited in predicting prognosis of NPC patients treated with two cycles of concurrent chemoradiotherapy. It is not adequate for other modalities such as induction chemotherapy or adjuvant chemotherapy, which require further investigation to develop new nomograms.

## Conclusions

We have established and validated nomograms that show higher predictive accuracy and independent discriminative ability, for OS and PFS of patients with NPC, compared to the TNM staging system. Furthermore, risk stratification allows clinical practitioners to identify prognosis of individual patients. Moreover, we suggest that GTV and CNV could improve the ability for the prognosis of T- and N-stages. GTV and CNV should be included in the TNM staging system.

## Data Availability Statement

The data of our study was derived from patients’ clinical information. To protect their privacy, we can’t upload the dataset. Requests to access the datasets should be directed to: C-DZ, zcd159179@163.com.

## Ethics Statement

The studies involving human participants were reviewed and approved by the Department of the Ethics Committee, Cancer Hospital of Shantou University Medical College. Written informed consent to participate in this study was provided by the participants’ legal guardian/next of kin. Written informed consent was obtained from the individual(s), and minor(s)’ legal guardian/next of kin, for the publication of any potentially identifiable images or data included in this article.

## Author Contributions

C-DZ and Z-NY contributed to conception and design of the study. C-DZ, Z-MC and Y-JH organized the database. C-DZ performed the statistical analysis. C-DZ wrote the first draft of the manuscript. ML, Y-JH, Z-MC, K-CH and Z-XL wrote sections of the manuscript. All authors contributed to the article and approved the submitted version.

## Conflict of Interest

The authors declare that the research was conducted in the absence of any commercial or financial relationships that could be construed as a potential conflict of interest.
