# Supplementary material for: Development and Validation of Prognostic Nomograms Based on Gross Tumor Volume and Cervical Nodal Volume for Nasopharyngeal Carcinoma Patients With Concurrent Chemoradiotherapy
Source: Front Oncol. 2021 Jun 28;11:682271. doi: 10.3389/fonc.2021.682271 (PMC8273655; doi:10.3389/fonc.2021.682271)
Supplement: Supplementary file 1 [file Table_1.docx]

Supplementary Material

**Supplementary table**

**Table S1│**Univariate cox regression analysis for OS and PFS in NPC patients in the training set

| Variables | OS | | | PFS | | |
| --- | --- | --- | --- | --- | --- | --- |
|  | P values | HR | 95% CI | P values | HR | 95% CI |
| GTV (ml) |  |  |  |  |  |  |
| ≤24.9 | 0.000 |  |  | 0.000 |  |  |
| >24.9,≤58.9 | 0.000 | 0.291 | 0.165 to 0.514 | 0.000 | 0.416 | 0.272 to 0.635 |
| >58.9 | 0.133 | 0.672 | 0.401 to 1.128 | 0.009 | 0.554 | 0.355 to 0.863 |
| CNV (ml) |  |  |  |  |  |  |
| ≤10.9 | 0.000 |  |  | 0.000 |  |  |
| >10.9，≤41.4 | 0.000 | 0.256 | 0.140 to 0.465 | 0.000 | 0.349 | 0.217 to 0.563 |
| >41.4 | 0.001 | 0.389 | 0.228 to 0.664 | 0.000 | 0.425 | 0.270 to 0.669 |
| GD (cm) |  |  |  | _ | _ | _ |
| ≤2.2 | 0.011 |  |  | _ | _ | _ |
| >2.2,≤4.0 | 0.003 | 0.376 | 0.198 to 0.713 | _ | _ | _ |
| >4.0 | 0.102 | 0.609 | 0.336 to 1.103 | _ | _ | _ |
| Age (years) |  |  |  |  |  |  |
| ≤51 | 0.000 |  |  | 0.011 |  |  |
| > 51,≤62 | 0.000 | 0.301 | 0.167 to 0.542 | 0.002 | 0.467 | 0.288 to 0.758 |
| >62 | 0.144 | 0.643 | 0.356 to 1.162 | 0.080 | 0.628 | 0.373 to 1.058 |

(Continued to the next page)

**Table S1│**Continued

| Variables | | OS | | | PFS | | |
| --- | --- | --- | --- | --- | --- | --- | --- |
|  |  | P values | HR | 95% CI | P values | HR | 95% CI |
| KPS | 0.001 | 0.927 | 0.885 to 0.971 | 0.000 | 0.930 | 0.895 to 0.965 |  |
| Tonsil | _ | _ | _ | 0.037 | _ | _ |  |
| Intraocluar muscle | 0.000 | 38.043 | 4.497 to 292.572 | 0.000 | 38.140 | 4.959 to 293.313 |  |
| Alveolar bone | 0.003 | 8.245 | 2.018 to 33.681 | 0.016 | 5.535 | 1.367 to 22.413 |  |
| Retropharyngeal space | 0.005 | 7.381 | 1.806 to 30.170 | 0.020 | 5.297 | 1.307 to 21.466 |  |
| Prevertebral space | 0.000 | 38.043 | 4.947 to 292.572 | 0.003 | 20.559 | 2.771 to 152.522 |  |
| Medial pterygoid | 0.002 | 2.001 | 1.286 to 3.114 | _ | _ | _ |  |
| Bone of skull base | 0.003 | 2.262 | 1.324 to 3.866 | 0.026 | 1.541 | 1.054 to 2.252 |  |
| Cervical vertebra | 0.025 | 2.808 | 1.135 to 6.943 | _ | _ | _ |  |
| Petrous bone | 0.015 | 1.741 | 1.115 to 2.717 | _ | _ | _ |  |
| Clivus | 0.011 | 1.768 | 1.138 to 2.747 | _ | _ | _ |  |
| Occipital bone | _ | _ | _ | 0.032 | 1.749 | 1.049 to 2.918 |  |
| Paranasal sinus | 0.036 | 1.651 | 1.034 to 2.638 | 0.007 | 1.671 | 1.149 to 2.430 |  |
| Eethmoidal sinus | 0.026 | 2.204 | 1.101 to 4.414 | _ | _ | _ |  |
| Cavernous sinus | 0.005 | 2.251 | 1.283 to 3.949 | 0.001 | 2.239 | 1.412 to 3.550 |  |
| Intracranial involvement | 0.034 | 1.811 | 1.046 to 3.135 | 0.003 | 1.936 | 1.248 to 3.002 |  |

(Continued to the next page)

**Table S1│**Continued

| Variables | OS | | | PFS | | |
| --- | --- | --- | --- | --- | --- | --- |
|  | P values | HR | 95% CI | P values | HR | 95% CI |
| Cranial nerve | 0.000 | 3.455 | 2.062 to 5.790 | 0.000 | 2.606 | 1.657 to 4.100 |
| External structure of lateral pterygoid | 0.034 |  |  | _ | _ | _ |
| Carotid sheath | 0.001 | 2.924 | 1.546 to 5.533 | 0.016 | 2.086 | 1.150 to 3.785 |
| Foramen   lacerum | 0.028 | 1.701 | 1.060 to 2.729 | _ | _ | _ |
| Ia-LN | _ | _ | _ | 0.001 | 28.475 | 3.776 to 214.717 |
| Ib-LN | 0.031 | 2.237 | 1.077 to 4.645 | 0.029 | 1.991 | 1.072 to 3.687 |
| III-LN | 0.021 | 1.684 | 1.081 to 2.624 | 0.040 | 1.436 | 1.016 to 2.030 |
| IV-LN | 0.000 | 3.174 | 1.782 to 5.654 | 0.000 | 3.188 | 1.974 to 5.150 |
| Va-LN | 0.044 | 1.667 | 1.013 to 2.743 | 0.006 | 1.727 | 1.167 to 2.556 |
| Vb/c-LN | 0.000 | 4.086 | 2.160 to 7.730 | 0.002 | 2.596 | 1.431 to 4.711 |
| VIII-LN | 0.034 |  |  | 0.007 | 4.852 | 1.539 to 15.300 |
| III-BLN | _ | _ | _ | 0.033 | 1.681 | 1.043 to 2.710 |
| IV-BLN | 0.011 | 4.454 | 1.404 to 14.124 | 0.000 | 6.222 | 2.527 to 15.324 |
| VIII-BLN | 0.001 | 30.339 | 4.008 to 229.678 | 0.002 | 23.888 | 3.198 to 178.444 |
| LNBC | 0.000 | 3.488 | 2.063 to 5.899 | 0.000 | 3.219 | 2.076 to 4.993 |
| BLNBC | 0.001 | 5.245 | 1.917 to 14.351 | 0.000 | 6.371 | 2.784 to 14.583 |

Bold text: statistical significance. OS, overall survival; PFS, progression-free survival; NPC, nasopharyngeal carcinoma; CI, confidence interval; GTV, gross tumor volume; CNV, cervical nodal volume; GD, the greatest dimension of cervical lymph nodes; Ia-LN, lymph node laterality metastasis of level Ia; Ib-LN, lymph node laterality metastasis of level Ib; III-LN, lymph node laterality metastasis of level III; IV-LN, lymph node laterality metastasis of level IV; Va-LN, lymph node laterality metastasis of level Va; Vb/c-LN, lymph node laterality metastasis of level Vb/c; VIII-LN, lymph node laterality metastasis of level VIII; III-BLN, bilateral lymph node metastasis of level III; IV-BLN, bilateral lymph node metastasis of level IV; VIII-BLN, bilateral lymph node metastasis of level VIII; LNBC, positive cervical lymph node laterality below the caudal border of cricoid cartilage; BLNBC, positive bilateral cervical lymph node below the caudal border of cricoid cartilage.
